# Bacteriologic study of cirrhotic patients with non-neutrocytic ascites 

**Published:** 2014

**Authors:** Hossein Dabiri, Masoumeh Azimi Rad, Ramin Tavafzadeh, Effat Taheri, Soudabeh Safakar, Ehsan Nazemalhosseini Mojarad, Neda Farzaneh, Mohammad Reza Zali

**Affiliations:** 1 *Gastroenterology and Liver Diseases Research Center, Shahid Beheshti University of Medical Sciences, Tehran, Iran *; 2 *Department of Medical Microbiology, Faculty of Medicine, Shahid Beheshti University of Medical Sciences, Tehran, Iran *; 3 *Psychiatry Department, School of Medicine, University Social Welfare and Rehabilitation Sciences, Tehran, Iran*; 4 *Emam Hossein Hospital, Shahid Beheshti University of Medical Sciences, Tehran, Iran*

**Keywords:** Ascitic fluid, BactDNA, Cirrhosis, Culture-negative non-neutrocytic ascites, Spontaneous bacterial peritonitis

## Abstract

**Aim:** We aimed for detection of bacterial DNA (bactDNA) in spontaneous bacterial peritonitis (SBP) by polymerase chain reaction (PCR) and its prognostic relevance in cirrhotic patients with culture-negative non-neutrocytic ascites (CNNNA).

**Background:** approximately 60% of patients with spontaneous bacterial peritonitis (SBP) are ascites culture negative.

**Patients and methods: **Of each 77 patients with cirrhosis and ascites, two samples including blood and ascitic fluid (AF) were taken. Blood samples were obtained for routine biochemical study and PMN count. AF samples were used for biochemical analysis and aerobic and anaerobic culture. BactDNA was detected by polymerase chain reaction (PCR) using bacterial universal 16srRNA gene primer.

**Results:** Of all AF samples, 3 (3.9%) were positive for bacterial culture (one *streptococcus α hemolytic* and two *E.coli)*. The mean number of PMN in AF was 63. BactDNA was detected in 33 (42.9%) of 77 of samples (group A) and bactDNA was absent in 41 (53.2%) of samples (group B). Blood WBC, prothrombin time, LDH, serum total protein, AF WBC, serum albumin, AF albumin, AF total protein, serum total bilirubin, AST, ALT and BUN were not statically different among group A and B. Hepatitis B, 41(45%), was the most frequent cause of cirrhosis.

**Conclusion:** Hepatitis B is the common cause of cirrhosis in Iranian cirrhotic patients. Also, current study showed that high number of Iranian cirrhotic patients with CNNNA carries bactDNA in their AF. The clinical findings as well as clinical laboratory data in patients with CNNNA are independent to bactDNA status in their ascitic fluid.

## Introduction

 Ascites is called for accumulation of fluid in the abdominal cavity, which is common in people with cirrhosis. Ascites usually develops when the liver is starting to fail. In general, the development of ascites indicates advanced liver dysfunction and patients should be referred for consideration of liver transplantation. The development of ascites is the most common complication among the estimated 3 million individuals with cirrhosis in the United States. Patients with cirrhosis and ascites carry a 10% annual risk of ascitic fluid infection (1). Spontaneous bacterial peritonitis (SBP) is a life threatening complication that arises almost exclusively in patients with cirrhosis (2). It has been described as an ascitic fluid (AF) infection in the absence of any intra-abdominal, surgically treatable source of infection (3). SBP is diagnosed on basis of polymorphonuclear (PMN) cell count) greater than 250cell/microlitre in ascites culture (4). Some patients have a clinical picture of SBP and elevated PMN count in ascites but negative ascitic fluid culture. This picture has been called, culture-negative neutrocytic ascites (CNNA) **(**5,6**)**, probable SBP (7,8). However, death was encountered less frequent in patients with culture-negative neutrocytic ascites than in those with culture-positive SBP (50% and 70%, respectively). The course of culture-negative neutrocytic ascites seemed to be less severe than those of culture-positive SBP (5). However, in general practice, ascites culture is negative in approximately 60% of patients with SBP (2,9-11). In a study between all bacterial infections diagnosed in patients with cirrhosis in a liver unit between 1998 and April 2000, gram-positive cocci were responsible for 53% of total bacterial infections (11). Increasing evidences point to a key role for bacterial translocation of intestinal flora from the intestinal lumen in combination with failure of anti-bacterial defense mechanisms to efficiently clear these translocating microorganisms (12). Gram-negative members of the Enterobactereaceae family (such as *Escherichia coli* and *klebsiella *spp.), enterococci and other streptococci species are the most effective at bacterial translocation to mesenteric lymph nodes (13). Clinical studies of bacterial translocation in cirrhosis have been limited by the lack of non-invasive methods to detect its presence (14). The presence of bacterial DNA (bactDNA) in serum and ascitic fluid has been proposed as a marker of bacterial translocation (15). Using such molecular techniques, bacterial translocation may be present in as many as one-third of cirrhotic patients with non-neutrocytic and culture-negative ascites; with *E.coli* the most frequently identified bacterial species (15). To our knowledge, no study about bacterial prevalence of a microorganism in cirrhotic patients has been done in Iran. Therefore, the main aim of the present study was to evaluate 16s rRNA gene amplification for diagnosis of ascites samples in cirrhotic patients with non-neutrocytic ascites samples. Supposing that according to literature review, Jose Such and Ruben Frances (15) suggest that even in cases of bacterial opsonization, rests of bacterial wall or inner components of bacteria might remain in the biological fluid. According to this hypothesis, we should be able to detect the passage of bacteria in blood or AF (16). Thus, the present study planned to detect minimal amounts of bactDNA in AF from cirrhotic patients with culture-negative non-neutrocytic ascitic fluid (CNNNA). 

## Patients and Methods

**Studied population**

From May 2012 to May 2013, 78 consecutively admitted patients with cirrhotic and ascites referred to Taleghani General Hospital, Tehran, Iran were enrolled in this cross-sectional study. Cirrhosis was diagnosed by patient’s clinical history, laboratory and ultra sonographic findings. Exclusion criteria were presence of culture-positive AF, the cases with non-cirrhotic ascites, the ascitic fluid total protein concentration greater than 2, SAAG≤1.1 (serum-to-ascites albumin gradient), neutrocytic AF (≥250 polymorphonuclear/ml) and intake of antibiotic within the previous two weeks. Simultaneously to sampling, therapeutic procedure was performed in all patients at the time of admission under aseptic conditions following the usual procedures. Informed consent was obtained from all patients, and the ethical committee of Research Center approved the protocol.

**Biochemical analysis**

 Two samples were obtained from each patient, blood and ascites fluid. Blood was investigated for total bilirubin, serum albumin, prothrombin time (PT), blood WBC, serum creatinine, and serum total protein by Roche Hitachi 717 Chemistry Analyzer (Japan). Simultaneously, a paracentesis was performed in case of all patients at admission in aseptic conditions. AF samples were transported to laboratory by heparin tube (pyrogen free tube) for molecular techniques and EDTA for PMN count and lactate dehydrogenase (LDH), total protein, and albumin, respectively. Routine biochemical study for AF samples was performed by Roche Hitachi 717 Chemistry Analyzer (Japan). PMN was counted by using Neobar slide method by skilled hematologist and then were checked by cell counter (Sysmex Kx-21, UK).

**Bacterial culture**

Besides, 3-5ml of AF were transported into screwed tubes contain 10ml of thioglycolate broth and were been incubated for 7 days at 37°C. Every 48 hours, one milliliter of thioglycolate were transported to Brucella Agar (Merck, Germany) with vitamin k (sigma Aldrich) for analysis of anaerobic organisms. In case of aerobic organism investigation, one milliliter of AF was directly cultured on the MacConkey Agar (Merck, Germany), Blood Agar and was evaluated for any growth after 24 hours. 

**DNA extraction and PCR**

DNA from each AF was extracted by using commercially available kit (Qiagen, Hilden, Germany). Presence of bacterial DNA was assessed by using universal bacterial 16s rRNA primer, 5’-AGA GTT TGA TCC TGG CTC AG- 3’ and 5’-ACG GCT ACC TTG TTA CGA CTT-3’, which amplify approximately 1500 bp fragment (17,18). All PCR mixtures were prepared in a volume of 25µL containing 1×PCR buffer, 500nM of each primer, 1.5mM MgCl_2_; 200µM each dNTP, 1.5U Taq DNA polymerase, and 300ng DNA sample. The mixtures were placed in a thermocycler (Eppendorf AG 22331, Hamburg, Germany), the PCR temperature condition was an initial denaturation step at 94°C for 4min; followed by 35 cycles, each of a denaturation step at 94°C for one minute, a primer-annealing step at 60°C for 30s, and an extension step at 72°C for 1.5 min; and a final step of 72°C for 10 min. PCR products were visualized by electrophoresis in 1.2% agarose gel, stained with ethidium bromide, and examined under UV illumination. 

**Statistical analysis**

Data processing and analysis were performed with SPSS software (version 13.0). Comparisons between groups were performed with *t*-test for quantitative data and with Fisher’s exact test for qualitative data. *P *value <0.05 and 2-sided were considered to indicate statistical significance. 

## Results

Considering exclusion and inclusion criteria, of 77 patients, 3 patients with culture positive for bacteria were excluded from study. Seventy-four consecutively admitted patients; 53(71.6%) male and 21(28.4%) female, mean age 54.1±16.6 with cirrhosis based on the inclusion and exclusion criteria as described above were included in this present cross-sectional study. Out of all patients with non-neutrocytic, 3(4%) patients have culture positive AF, of these three culture positive samples, from one sample *streptococcus α hemolytic *and from other two culture positive *E.coli *were identified. Therefore, two of these patients, were HbsAg positive. BactDNA was positive in all 3 cultures positive samples. The three culture positive samples were excluded from study according to study criteria. Overall, regardless to bactDNA status the mean number of PMN in AF was 63ml-1 (Interquartile range: 8-100ml-1). Patients with negative culture were classified into two groups; A and B according to the presence (group A) or absence (group B) of bacterial DNA in AF. Out of 74 cultures negative AF, 33 (45%) samples bactDNA were detected (Group A), while 41 (55%) samples were negative for bactDNA (Group B). Clinical and laboratory features in patients with CNNNA (culture-negative non-neutrocytic ascites) with (group A) and without (group B) positive bactDNA are summarized in Tables 1 and 2. Demographic data including age, sex did not vary statically in both groups (*P*>0.05). Blood WBC, prothrombin time, LDH, Serum total protein and AF WBC were higher in group A (with positive bactDNA), while serum albumin, AF albumin, AF total protein, serum total bilirubin, AST, ALT and BUN were lower than group B (*P*<0.05). The comparison of clinical items including fever, abdominal pain, tenderness and chill did not reach to statistical significance in groups A and B (Table 1). Based on our data, hepatitis B was the most frequent cause of cirrhosis [41 (45%)], followed by hepatitis C [14 (18%)], autoimmune hepatitis [8 (10%)], hepatitis D/hepatitis B [1 (1%)] and cirrhosis of unknown origin [13 (17%)]. The distribution of hepatitis B and C were 42% and 21% in group A, and 46% and 17% for group B, respectively. Of all studied patients, only one patient in group B was diagnosed with hepatitis D/B. Autoimmune hepatitis was more common in group B (19.5%) compare to group A (3%) (*P*<0.004) (Table 1). 

**Table 1:** Distribution of main demographic features and clinical finding among patients with non- neutrocytic ascites*

Feature		Group A (33)	Group B (41)	Culture positive (3)	Total (77)
Age		56.4±15.8	52.6±16.5	55±8.2	54.3 ±16.2
Sex	Male	25(76%)	28(68%)	3(100%)	56(72.7%)
	Female	8(24%)	13(32%)	0	21(27.3%)
Cause of cirrhosis	Hepatitis B	14(42%)	19(46.3%)	2 (66.6%)	35(47%)
	Hepatitis C	7(21.2%)	7(17%)	0	14(19%)
	Auto- immune Hepatitis	1(3%)	8(19.5%)	0	9(12%)
	Hepatitis D/ Hepatitis B	0	1(2.4%)	0	1(1%)
Chill		5 (51%)	8 (54% )	--	13(17%)
Tenderness		10 (33% )	13(36% )	--	23(30%)
Abdominal pain		23 (53%)	28(36%)	2(66.6%)	53(68.8%)
Fever		11 (26%)	16(29% )	1	28

* Group A: culture- negative non- neutrocytic ascites with positive bactDNA, Group B: culture-negative non-neutrocytic ascites with no bactDNA, AF: ascitic fluid

**Table 2:** Distribution of laboratory finding among patients with culture-negative non-neutrocytic ascites*

Laboratory finding	Group A (33)	Group B (41)	P-value
AF-PMN/ mm^3^	61.3±22	66.8±22	0.29
AF WBC/mm^3^	386±134	956±341	0.15
PT (second)	18.5±1.6	16.8±0.6	0.27
AF- LDH(U/L)	216.8±69.9	388.8±175.2	0.40
Serum albumin (gr/dl)	2.98±0.24	4.6±1.7	0.40
AF- albumin (gr/dl)	1.6 ±0.2	2.8±1.4	0.44
AF - total protein (gr/dl)	39.8±26.4	21.5±18.6	0.56
Serum total protein (gr/dl)	5.6±0.4	23±16.9	0.35
Total bilirubin (mg/dl)	3.1±1.4	4.9±1.8	0.44
Creatinine (mg/dl)	1.77±0.4	1.95±0.4	0.75
BUN(mg/dl)	35±4.75	41.5±5.5	0.39
AST(U/L)	130±79.8	82±24	0.53
ALT(U/L)	31.6±3.46	57.1±18.9	0.24

* AF: Ascitic fluid, PMN: polymorphonuclear , Group A: culture- negative non- neutrocytic ascites with positive bactDNA, Group B: culture- negative non- neutrocytic ascites with no bactDNA, PT: Prothrombin Time, LDH: Lactate dehydrogenase, BUN: Blood Urea Nitrogen, AST: Aspartate aminotransferase, ALT: Alanine transaminase

## Discussion

 Recent investigations have shown that the presence of bactDNA in blood and AF constitutes an independent predictor of mortality in patients with cirrhosis (17). The mortality rate significantly increases in bactDNA (+) patients, and acute-on-chronic liver Failure (AOCLF) is the most frequent cause of death related to bactDNA presence in the first month after inclusion (17). To our knowledge, this study is the first to evaluate the prevalence of bacterial DNA in non-neutrocytic ascites by polymerase chain reaction (PCR) in Iran. Although in Iran physicians did not commonly ask laboratory to perform AF bactDNA for patients with ascites. However, this method is available in remarkable numbers of laboratories. Previous investigations around the world have showed that approximately 14% to 32% of cirrhotic patients with culture-negative non-neutrocytic ascitic fluid (CNNNA) have episodes of bactDNA translocation (4,18). Our study showed that bactDNA was positive in 45% of culture-negative non-neutrocytic ascitic fluid (CNNNA) of cirrhotic patients. Compared with other results, the higher prevalence in our study may be explained primarily, by differences in the patient population (presence of an inflammatory state, antibiotic pretreatment) and by differences in the analytical sensitivity of the assays (4). Since the same physician under the usual conditions of sterility has performed all samples, thereby contamination is not considered as a reason for high incidence of bactDNA in our CNNNA samples. This finding showed that spontaneous bacterial peritonitis (SBP) is a more threatening complication for Iranian patients with cirrhosis. Comparison of two CNNNA patient groups with (A group) and without (B group) positive bactDNA showed, serum creatinine and AF PMN had no obvious differences. However, the AF PMN was slightly higher in bactDNA negative patients (Table 1). Distribution of viral hepatitis (HBV, HBC, and HDV/HBV) was similar in both group, so probably we could conclude viral hepatitis do not promote BSP. In addition, abdominal pain, tenderness, fever and chill did not differ statically in both groups (*P*>0.05). Therefore, our result indicates that the presence of bactDNA in ascites is not associated with these clinical items neither AF PMN count. According to laboratory finding none of investigated parameters including AF WBC, PT (second), AF- LDH, serum albumin, AF-albumin, AF-total protein, serum total protein, total bilirubin, creatinine, BUN, AST, ALT levels did not distinguish patients with culture-negative non- neutrocytic ascites with and without bactDNA. In non-neutrocytic ascites from patients with end-stage liver disease and in animal model, bactDNA from the following gram-positive and gram-negative organisms has been isolated: *E. coli*, *S. aureus* and *Staphylococcus *spp*.*, *Klebsiella *spp., *Enterococcus *spp., and *Enterobacter *spp. are the most isolated bacteria (11-14,18). In the current study, the isolation of *E.coli* and *Staphylococcus *spp. in very limited culture positive samples is compatible with previous reports. We could not find any statically association among clinical and para-clincal data with the culture result. This may contribute to the very low number of culture positive sample. 

In conclusion, in respect to high presence of bactDNA in CNNNA patients with cirrhosis using high sensitive PCR method in Iran, the threat of BSP and death is an increasing concern, especially in developing countries where infection control remain a major challenge. However, more and continually studies are needed to ascertain the precise association of clinical signs and symptoms with laboratory findings.
